# Cardiology clinic visit increases likelihood of evidence‐based cholesterol prescribing in severe hypercholesterolemia

**DOI:** 10.1002/clc.23521

**Published:** 2020-12-23

**Authors:** Nicole A. Groth, Neil J. Stone, Catherine P. Benziger

**Affiliations:** ^1^ Essentia Institute of Rural Health Duluth Minnesota USA; ^2^ Feinberg School of Medicine Northwestern University Chicago Illinois USA; ^3^ Essentia Health Heart and Vascular Center Duluth Minnesota USA

**Keywords:** atherosclerotic cardiovascular disease, guideline adherence, low‐density lipoprotein cholesterol, rural healthcare, severe hypercholesterolemia

## Abstract

**Background:**

Patients with phenotypic severe hypercholesterolemia (SH), low‐density lipoprotein‐cholesterol (LDL‐c) ≥ 190 mg/dl, atherosclerotic cardiovascular disease (ASCVD) or adults 40–75 years with diabetes with risk factors or 10‐year ASCVD risk ≥20% benefit from maximally tolerated statin therapy. Rural patients have decreased access to specialty care, potentially limiting appropriate treatment.

**Hypothesis:**

Prior visit with cardiology will improve treatment of severe hypercholesterolemia.

**Methods:**

We used an electronic medical record‐based SH registry defined as ever having an LDL‐c ≥ 190 mg/dl since January 1, 2000 (*n* = 18 072). We excluded 3205 (17.7%) patients not alive or age 20–75 years. Patients defined as not seen by cardiology if they had no visit within the past 3 years (2017–2019).

**Results:**

We included 14 867 patients (82.3%; mean age 59.7 ± 10.3 years; 58.7% female). Most patients were not seen by cardiology (*n* = 13 072; 72.3%). After adjusting for age, sex, CVD, hypertension, diabetes and obesity, patients seen by cardiology were more likely to have any lipid‐lowering medication (OR = 1.46, 95% CI: 1.29–1.65), high‐intensity statin (OR = 1.81, 95% CI: 1.61–2.03), or proprotein convertase subtilisin‐kexin type 9 (PCSK9) inhibitor (OR = 5.96, 95% CI: 3.34–10.65) compared to those not seen by cardiology. Mean recent LDL‐c was lower in patients seen by cardiology (126.8 ± 51.6 mg/dl vs. 152.4 ± 50.2 mg/dl, respectively; *p* < .001).

**Conclusion:**

In our predominantly rural population, a visit with cardiology improved the likelihood to be prescribed any statin, a high‐intensity statin, or PCSK9 inhibitor. This more appropriately addressed their high life‐time risk of ASCVD. Access to specialty care could improve SH patient's outcomes.

**Abbreviations**
ACC: American College of Cardiology
AHA: American Heart Association
ASCVD: atherosclerotic cardiovascular disease
CVD: cardiovascular disease
EAS: European Atherosclerosis Society
EH: Essentia Health
ESC: European Society of Cardiology
HDL‐c: high‐density lipoprotein cholesterol
LDL‐c: low‐density lipoprotein cholesterol
PCSK9: proprotein convertase subtilisin‐kexin type 9
SH: severe hypercholesterolemia

## 1 INTRODUCTION

Cardiovascular disease (CVD) is the leading cause of death in America and across the globe^1^, ^2^, ^3^, ^4^ and coronary artery disease is the most prevalent type of CVD. Nearly half (49%) of all Americans have one of the following risk factors for heart disease: elevated blood pressure, elevated low‐density lipoprotein cholesterol (LDL‐c), or smoking.^5^ Elevated LDL‐c, or severe hypercholesterolemia (SH), is defined as an LDL‐c > 190 mg/dl. Patients with evidence of SH are at increased risk for atherosclerotic cardiovascular disease (ASCVD) and roughly 600 000 people in the United States manifest the phenotype.^6^ The American Heart Association/American College of Cardiology (AHA/ACC) goals for 2019 focused on the primary prevention of CVD. One of the top 10 take‐home messages for primary prevention of CVD in the 2018 guideline on cholesterol management recommends patients with primary SH be started on a maximally tolerated statin therapy without further risk stratification.^7^ Despite the overwhelming evidence for statin therapy, there is still a large divide in patients who are eligible for and recommended by the national guidelines to be on a statin and those who are actually receiving statin therapy.^8^, ^9^, ^10^, ^11^

Patients presenting with evidence for SH along with other comorbidities, including diabetes, cigarette smoking, and hypertension, are at increased risk for CVD.^12^ Management of these comorbidities could help reduce the risk for CVD in patients with a high LDL‐c.^13^ While patients with multiple comorbidities are more likely to be seen by a physician, this surprisingly has little impact on the likelihood of a patient receiving guideline‐directed cholesterol management to reach their LDL‐c goals.^14^ Prior studies showed significant increase in statin adherence for patients with a higher number of lipids panels completed, and therefore more visits to their doctor's office.^15^

One barrier to health equity (i.e., guideline‐directed cholesterol management) within the Essentia Health (EH) population is service area. While about 20% of the United States population resides in rural area, very few specialty clinics exist within rural communities.^16^ EH encompasses a largely rural population which can make it difficult for patients to get access to specialty‐care providers, like cardiology. Distance from a specialty clinic forces rural patients to commute significant lengths for focused and advanced care. Commuting challenges result in approximately 3.6 million Americans, per year, missing or delaying medical appointments.^17^

The impact of being seen by cardiology on the use of lipid‐lowering medication is not well‐characterized.^9^ We aimed to evaluate use of statins in SH patients in our predominantly rural population, based on prior visit with cardiology.

## 2 METHODS

This is a cross‐sectional study of patients who were in an electronic medical record‐based SH registry defined by ever having an LDL‐c > 190 mg/dl between January 1, 2000–June 1, 2020 (*n* = 18 072) at EH. EH is an integrated healthcare delivery system with facilities in four states (Minnesota, Wisconsin, North Dakota, and Idaho) that integrates physician group practice, acute care delivery including primary, secondary, and tertiary care centers, critical access hospitals, long‐term care facilities, hospice care, medical equipment, and ambulance services. EH services cover an area of approximately 55 000 sq. miles with over 1 million residents. Within this area, EH has 15 hospitals, 74 clinics, and 1700 physicians and credentialed practitioners with approximately 65% of the patients served living in rural areas. The study was approved by the Institutional Review Board of Essentia Health. The need to obtain informed consent was waived for the collection, analysis and publication of the retrospectively obtained and anonymized data for this non‐interventional study.

**FIGURE 1:**
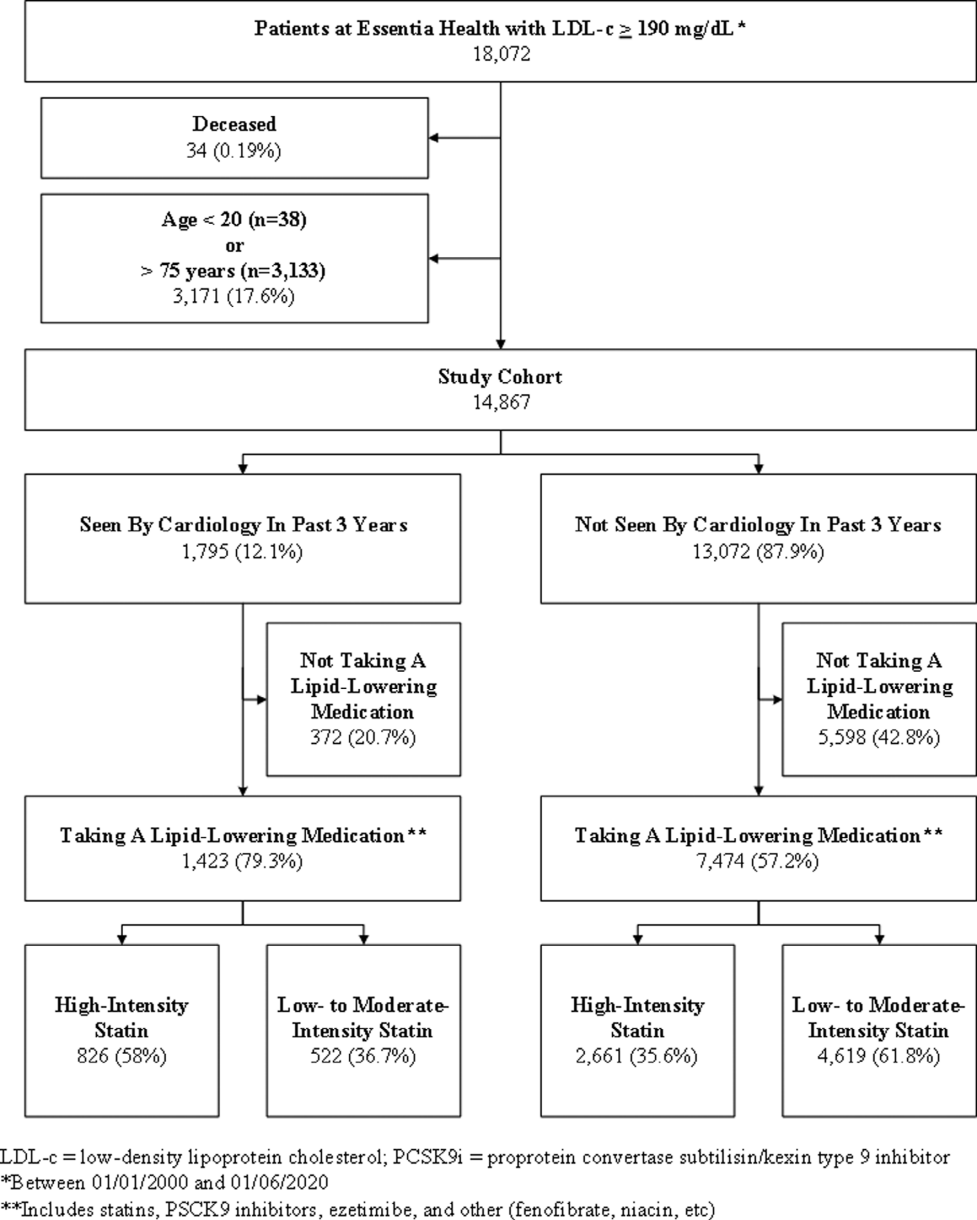
Flow chart of patient cohort from the electronic medical record‐based severe hypercholesterolemia registry defined for patients who had ever had an LDL‐c ≥ 190 mg/dl between January 1, 2000 and June 1, 2020 (*n* = 18 072)

Patients were excluded if they were age <20 years (*n* = 38) or >75 years (*n* = 3133), due to inconsistency in the applicability of the evidence‐based intervention suggested in the cholesterol guidelines^7^ outside of that age range. Deceased patients were also excluded from analysis (*n* = 34; 0.19%; Figure 1). Patients were considered to have not been seen by cardiology if there was no documented clinic visit within the past 3 years. Majority of patients had been seen in primary care clinic in the past 5 years (98.8%). All visits were in person and not via telehealth during this study period.

Medications were pulled from the patient's active medication list. Only atorvastatin 40 mg or 80 mg or rosuvastatin 20 mg or 40 mg were considered high‐intensity statins. All other statin medications and doses were considered low‐ or moderate‐intensity. Due to effectiveness in LDL lowering, patients prescribed a proprotein convertase subtilisin‐kexin type 9 (PCSK9) inhibitor were grouped with the high‐intensity statin cohort, while patients prescribed Ezetimibe were combined with the low‐ to moderate‐intensity statin group. Statin allergies were obtained from the patient's allergy list.

Baseline risk factors were obtained from the problem list using ICD‐10 diagnoses, recent vitals, and laboratory results. Diabetes was defined as most recent glucose ≥126 mg/dl or A1c ≥ 6.5% or currently taking a glucose‐lowering medication. Patients who did not have lab values or medication information on file were compared against a diabetes registry and considered diabetic if they ever had an active status. Hypertension was defined as blood pressure ≥ 140/90 mm Hg or currently taking antihypertensive medication. Patients who did not have vitals or medication information on file were compared against a hypertension registry and considered to have hypertension if they ever had an active status. Obesity was defined as body mass index ≥30 mg/m^2^. Smoking status was asked at the most recent visit and defined as never smoker, former smoker (quit <12 months prior), and current smoker. Prior CVD was defined as ever having an ICD‐10 diagnosis of ASCVD on their problem list (full list of ICD‐10 codes available in Appendix A). Date and value of the highest and most recent total cholesterol, LDL‐c, high‐density lipoprotein cholesterol (HDL‐c), and triglycerides were recorded. Approximately 5% of the cohort had missing values for total cholesterol, HDL‐c, and triglycerides (*n* = 794; 5.3%, *n* = 779; 5.2%, *n* = 792; 5.3%, respectively).

Categorical variables were expressed as absolute numbers and percentages and compared with chi‐squared test. Continuous variables are expressed as means and SDs (median with 25th–75th percentile interquartile range for non‐normally distributed variables) and were compared using a *t*‐test where appropriate. Univariate and multivariate logistic regression models with 95% confidence intervals (CI) were calculated adjusting for age, sex, history of ASCVD, hypertension, obesity, and diabetes. A *p*‐value of <.05 was considered statistically significant. All statistical analyses were performed with STATA 16.0 (College Station, TX).

## 3 RESULTS

We identified 14 867 patients within the EH system with evidence for SH that did not meet exclusion criteria (58.7% female, mean age 59.7 + 10.3 years). Of these patients, 1795 (12.1%) had been seen by cardiology within the past 3 years, whereas 13 072 (87.9%) did not have a recent cardiology visit. Severe hypercholesterolemia patients who had been seen by cardiology had a higher prevalence of hypertension, diabetes mellitus, and coronary artery disease, compared with SH patients who had not been seen by cardiology (Table 1). Specifically, patients seen by cardiology had CAD (72.4 vs. 20.4%) and were current or former smokers (62.9 vs. 52.0%).

**TABLE 1:** Baseline demographics, smoking statistics, and clinical measurements of Essentia Health patients with clinical findings for severe hypercholesterolemia (low‐density lipoprotein cholesterol ≥190 mg/dl) since January 1, 2000 (*n* = 14 867)

	*N*	Seen by cardiology	Not seen by cardiology	*p*‐value
		*N* = 1795	*N* = 13 072	
**Demographics**				
Age, years^a^	14 867	63.4 ± 8.8	59.2 ± 10.4	<.001
20–39 years, *n* (%)		34 (1.9)	747 (5.7)	
40–49 years, *n* (%)		108 (6.0)	1517 (11.6)	
50–59 years, *n* (%)		371 (20.7)	3512 (26.9)	
60–75 years, *n* (%)		1282 (71.4)	7296 (55.8)	
Male, *n* (%)	14 867	846 (47.1)	5298 (40.5)	<.001
Prior ASCVD, *n* (%)	14 867	1300 (72.4)	2670 (20.4)	<.001
Diabetes, *n* (%)	14 867	442 (24.6)	1710 (13.1)	<.001
Hypertension, *n* (%)	14 867	619 (34.5)	2822 (21.6)	<.001
**Smoking status**	14 867			<.001
Current, *n* (%)		328 (18.3)	2209 (16.9)	
Former, *n* (%)		801 (44.6)	4584 (35.1)	
Never, *n* (%)		666 (37.1)	6279 (48.0)	
**Clinical measurements**				
Total cholesterol (mg/dL)^a^	14 073	206.3 ± 59.2	233.7 ± 55.7	<.001
LDL‐c (mg/dL)^a^	14 867	126.8 ± 51.6	152.4 ± 50.2	<.001
HDL‐c (mg/dL)^a^	14 088	49.5 ± 13.6	53.5 ± 14.7	<.001
Triglycerides (mg/dL)^a^	14 075	165.8 ± 109	159.1 ± 104.4	.015

Abbreviations: ASCVD, atherosclerotic cardiovascular disease; HDL‐c, high‐density lipoprotein cholesterol; LDL‐c, low‐density lipoprotein cholesterol.

^a^ Values are mean ± SD.

**FIGURE 2:**
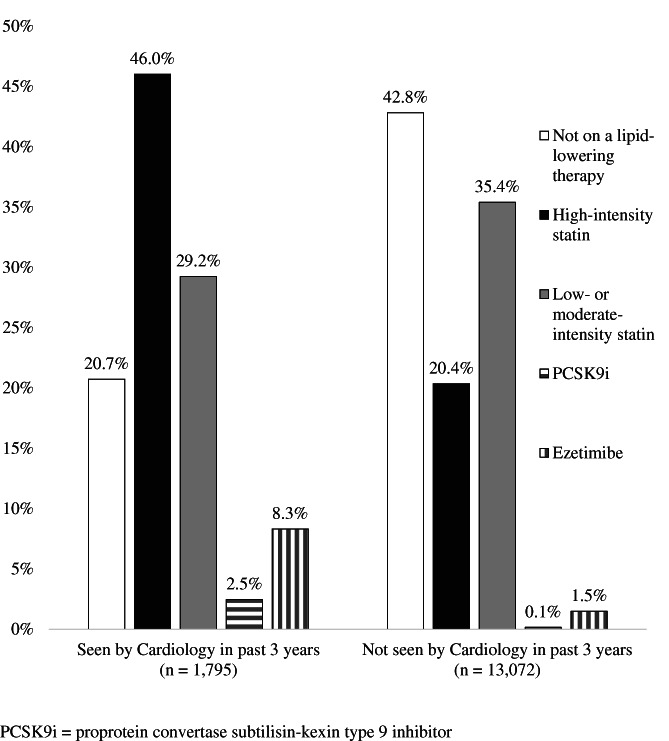
Results of a cross‐sectional cohort study, estimating statin utilization by intensity, from an electronic medical record‐based hyperlipidemia registry (*n* = 14 867; defined as ever having an LDL‐c ≥ 190 mg/dl since January 1, 2000)

In total, 8894 (59.8%) SH patients were prescribed a lipid‐lowering medication. A greater proportion of the patients who had been seen by cardiology (79.3%) were prescribed a lipid‐lowering medication compared to the group that had not been seen by cardiology (57.2%) (Figure 2). Also, a more significant number of patients seen by cardiology were prescribed a high‐intensity statin or PCSK9 inhibitor, compared to those not seen by cardiology (46.0 and 2.5 vs. 20.4% and 0.1%; *p* < .001). In the high‐risk subgroups, those with SH and diabetes (*n* = 2152) or hypertension (*n* = 3441), only 39.1% (*n* = 841) of the diabetics were prescribed a high‐intensity statin and approximately 28.5% (*n* = 981) of the hypertension patients had active statin prescriptions. Another high‐risk subgroup is the current smokers, of which 27.3% (*n* = 692 of 2537) were prescribed high‐intensity statin therapy.

After adjusting for age, sex, CVD, hypertension, obesity, and diabetes, SH patients who had been seen by cardiology had greater odds of being on any lipid‐lowering medication, high‐intensity statin, or PCSK9 inhibitor compared to those who had not been seen by cardiology (Table 2). When comparing patients without ASCVD (*n* = 495), those who had been seen by cardiology were more likely to be prescribed a statin compared to patients only seen by primary care (*n* = 10 402; 62.4 vs. 52.1%, p < 0.001). There was a significant difference between sex and lipid‐lowering medication use. Males were more likely than females to be prescribed any lipid‐lowering medication (OR 0.75, 95% CI: 0.71–0.81), high‐intensity statin (OR 0.30, 95% CI: 0.29–0.32), or PCSK9i (OR 0.53, 95% CI: 0.32–0.89).

**TABLE 2:** Comparison of lipid‐lowering prescription and lipid‐lowering intensity among patients with severe hypercholesterolemia, defined as low‐density lipoprotein cholesterol ≥190 mg/dL (between 01/01/2000 and 01/06/2020; *n* = 14 867)

	Cardiology Visit			
	No (*n* = 13 072)	Yes (*n* = 1795)	OR (95% CI) (unadjusted)	OR (95% CI) (adjusted^a^)
Receiving statin therapy	7289 (55.8%)	1351 (75.3%)	2.41 (2.16–2.70)	1.46 (1.29–1.65)
Receiving high‐intensity statin therapy	2661 (20.4%)	826 (46.0%)	3.34 (3.01–3.69)	1.81 (1.61–2.03)
Receiving PCSK9i therapy	19 (0.1%)	44 (2.5%)	17.26 (10.06–29.63)	5.96 (3.34–10.65)

Abbreviations: CI, confidence interval; PCSK9i, proprotein convertase subtilisin‐kexin type 9 inhibitor.

^a^ Adjusted for patient's age, sex, history of cardiovascular disease, hypertension, obesity, and diabetes.

**FIGURE 3:**
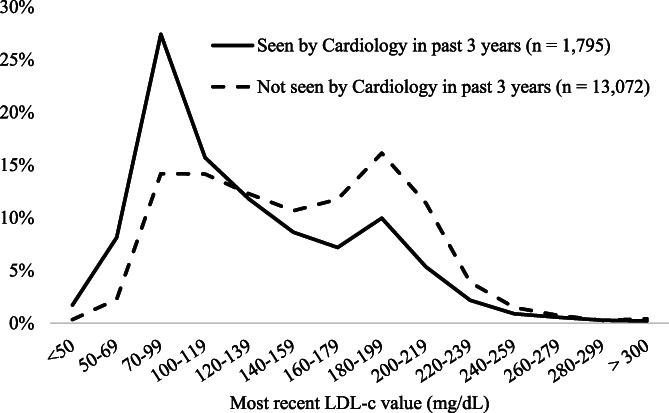
Most recent low‐density lipoprotein cholesterol (LDL‐c) value (mg/dL) in patients with severe hyperlipidemia (ever had an LDL‐c ≥ 190 mg/dl since January 1, 2000) at Essentia Health

Patients seen by cardiology had a significantly lower mean LDL‐c than those not recently seen by cardiology (126.8 + 51.6 mg/dl vs. 152.4 + 50.2 mg/dl; *p* < .001) (Table 1). About 38% (*n* = 683) of patients seen by cardiology had their LDL controlled to <100 mg/dl and 11% (*n* = 198) to <70 mg/dL compared to patients not seen by cardiology (17.6% (*n* = 2301) LDL‐c < 100 mg/dL and 2.9% (*n* = 380) LDL‐c < 70 mg/dl; Figure 3). Of the 372 patients seen by cardiology without a current lipid‐lowering medication prescription, 39.2% had a documented statin allergy or intolerance compared to 15.0% of the 5601 patients not seen by cardiology.

## 4 DISCUSSION

A large cohort of patients within the EH system met phenotypic criteria for SH and fewer than 13% of them had been seen by cardiology within the past 3 years. More alarming was that in this predominantly rural population, over 40% of patients with phenotypic SH do not have an active prescription for any lipid‐lowering medication. Our results show that patients who were seen by cardiology in the past 3 years were much more likely to be prescribed a lipid‐lowering medication (79.3 vs. 57.2%) and specifically a high‐intensity statin or PCKS9i compared with those who had not been seen by cardiology (46.0 and 2.5% vs. 20.4 and 0.1%, respectively). Patients who had been seen by cardiology were also more likely to have prior ASCVD, diabetes, hypertension, smoke, be female and of an older age than patients seen only by primary care. However, it is important to note, of the 17% of patients who saw cardiology that were for primary prevention, they were much more likely to be treated with a statin (62 vs. 52%).

Prior studies of patients with ASCVD in the Veterans Affairs system found similar results, showing a dose response relationship with patients having a higher statin use and statin adherence if they had more visits by either cardiology or primary care.^9^, ^18^ Other studies have noted routine, guideline‐directed completion of lipid panels is associated with a modest increase in statin adherence.^15^ Our study is unique in that it included not only patients with ASCVD but patients with phenotypic SH in a predominantly rural community. While we were not able to look at a dose response or adherence to statin medications, it is likely that cardiologists are more familiar with the cholesterol guidelines and are likely to check and treat cholesterol more aggressively than primary care.

The 2018 AHA/ACC^7^ and 2019 European Society of Cardiology and European Atherosclerosis Society (ESC/EAS)^19^ cholesterol guidelines recommend more aggressive lipid treatment than prior guidelines for the management of patients with phenotypic severe hypercholesterolemia, given that they are likely at increased risk of cardiac events due to ASCVD. We found that even after adjusting for baseline differences, patients who had been seen by cardiology had 46% higher odds of being prescribed any lipid‐lowering medication and 81% higher odds of being prescribed a high‐intensity statin, as well as six times higher odds of a prescription for PCSK9 inhibitor. These results are consistent with those previously observed in the Veterans Affairs Health Care System that showed a higher percentage of patients with ASCVD who saw cardiology had been prescribed high‐intensity statins.^9^

We found less than half of all patients with diabetes were on a high‐intensity statin and just over 63% of hypertension patients were receiving any intensity of statin therapy. This is slightly better than a previous study of a German diabetes registry which showed that of 51 640 SH patients with type 2 diabetes, 25.5% were prescribed a statin.^14^ A recent study at another large health system found that in 2017, 59.4% of patients with an LDL > 190 were on a statin and a percentage similar to our cohort were on ezetimibe (2.5%) and PCSK9 inhibitors (0.7%).^20^

Despite the overwhelming evidence for lipid‐lowering medication use, less than half of our patients with phenotypic SH were prescribed lipid‐lowering medication at a large healthcare system. Similar results have been seen in previous studies examining the use of lipid‐lowering medications.^8^, ^9^, ^21^, ^22^ Bradley C.K. et al.^8^ analyzed 5693 patients from the PALM registry (designed to evaluate lipid management practices as well as patient/provider beliefs on statin therapy) and found that 26.5% were not on treatment, while more than half of patients who were eligible for statin therapy but not taking any lipid‐lowering medication reported never being offered treatment. The trends in lipid‐lowering medication use between patients seen by cardiology and those not seen may provide some insight into why there is a such a discrepancy in treatment despite eligibility of the patients. A 2015 AHA/ACC national survey reported that 22% of the primary care providers (compared to 33% of specialists, including cardiology and endocrinology) surveyed knew the definition of high‐, intermediate‐, and low‐intensity statin therapy.^23^ Results showing higher lipid‐lowering medication use in patients who have been seen by cardiology may suggest a persistent effort put forward by cardiology to re‐challenge with a statin or explore alternative agents to statins when one statin may have previously caused an allergy or intolerance to the patient.^10^ In the future, adoption of quality measures that includes either appropriate percent lowering of LDL‐cholesterol, such as 50% lowering, and/or specific targets, such as LDL‐c < 70 mg/dl, should be required for those with ASCVD or for higher risk primary prevention. Those at high‐risk need to engage in a risk discussion with their clinician regarding the potential for benefit versus risk for additional cholesterol lowering therapy, such as ezetimibe or PCSK9 inhibitors.^24^, ^25^, ^26^, ^27^, ^28^ Moreover, risk discussions should remind physicians to increase statin intensity as necessary for patients with a history of SH. Other options for assisting in the discrepancy between eligibility for and use of high‐intensity statins may include use of computer prompts based on diagnoses and increased use of support staff like pharmacists and nurses to educate patients about the importance of lowering their LDL‐c to goal through the use of lipid‐lowering medications.

## 5 LIMITATIONS

EH is a large accountable care organization in a rural area of the Midwest and besides a higher Native American population, is largely Caucasian and may not reflect demographics in all regions. The cardiology clinic is staffed with physicians as well as advanced practice providers. Any LDL‐c > 190 mg/dl defined SH and the authors were unable to exclude erroneous labs or elevated LDL‐c due to secondary etiologies. Data on medication prescriptions was used but adherence was not tracked in this retrospective study. Very few patients had genetic testing or detailed clinical examination to confirm Familial Hypercholesterolemia. Details of statin allergy or intolerance were not available. Another limitation includes not excluding patients with the common secondary causes of hyperlipidemia, such as thyroid‐related disease, certain medications, and nephrotic syndrome. Prior studies suggest that this is likely only 1–2% of this population.^20^

## 6 CONCLUSIONS

Being seen by cardiology is associated with increased statin prescriptions in phenotypic SH. Better access to specialty care may improve cholesterol management. Due to the largely rural EH service area, overcoming barriers for patients to be seen by specialty practice like cardiology, future telehealth or virtual visits may provide better access to cardiology and therefore improve the guideline‐directed use of lipid‐lowering medications in this population.

## DISCLOSURES

The authors have no financial disclosures to report.

Dr. Stone is vice‐chair of the 2018 AHA‐ACC‐MultiSociety Guidelines.

## Supporting information


**Appendix A** ASCVD definition

## References

[1] Heron M . Deaths: leading causes for 2016. Natl Vital Stat Rep. 2018;67(6):1‐77. 30248017

[2] Cardiovascular diseases (CVDs) . https://www.who.int/news-room/fact-sheets/detail/cardiovascular-diseases-(cvds); 2017. Accessed May 17, 2017.

[3] Mc Namara K , Alzubaidi H , Jackson JK . Cardiovascular disease as a leading cause of death: how are pharmacists getting involved? Integr Pharm Res Pract. 2019;8:1‐11. 30788283 10.2147/IPRP.S133088 PMC6366352

[4] Murphy SL , Kochanek KD , Xu J , Arias E . Mortality in the United States, 2014. NCHS Data Brief. 2015;229:1‐8. 26727391

[5] Fryar CD , Chen TC , Li X . Prevalence of uncontrolled risk factors for cardiovascular disease: United States, 1999–2010. NCHS Data Brief. 2012;103:1‐8. 23101933

[6] Sniderman AD , Tsimikas S , Fazio S . The severe hypercholesterolemia phenotype: clinical diagnosis, management, and emerging therapies. J Am Coll Cardiol. 2014;63(19):1935‐1947. 24632267 10.1016/j.jacc.2014.01.060

[7] Grundy SM , Stone NJ . 2018 American Heart Association/American College of Cardiology/Multisociety guideline on the Management of Blood Cholesterol‐Secondary Prevention. JAMA Cardiol. 2019;4(6):589‐591. 30994869 10.1001/jamacardio.2019.0911

[8] Bradley CK , Wang TY , Li S , et al. Patient‐reported reasons for declining or discontinuing statin therapy: insights from the PALM registry. J Am Heart Assoc. 2019;8(7):e011765. 30913959 10.1161/JAHA.118.011765 PMC6509731

[9] Rehman H , Ahmed ST , Akeroyd J , et al. Relation between cardiology follow‐up visits, evidence‐based statin prescribing, and statin adherence (from the veterans affairs health care system). Am J Cardiol. 2019;124(8):1165‐1170. 31405545 10.1016/j.amjcard.2019.07.022

[10] Birtcher K . When compliance is an issue‐how to enhance statin adherence and address adverse effects. Curr Atheroscler Rep. 2015;17(1):471. 25410047 10.1007/s11883-014-0471-8

[11] Abul‐Husn NS , Manickam K , Jones LK , et al. Genetic identification of familial hypercholesterolemia within a single U.S. health care system. Science. 2016;354(6319):aaf7000. 28008010 10.1126/science.aaf7000

[12] Stone NJ , Levy RI , Fredrickson DS , Verter J . Coronary artery disease in 116 kindred with familial type II hyperlipoproteinemia. Circulation. 1974;49(3):476‐488. 4813182 10.1161/01.cir.49.3.476

[13] Perrot N , Verbeek R , Sandhu M , et al. Ideal cardiovascular health influences cardiovascular disease risk associated with high lipoprotein(a) levels and genotype: the EPIC‐Norfolk prospective population study. Atherosclerosis. 2017;256:47‐52. 27998826 10.1016/j.atherosclerosis.2016.11.010 PMC5321848

[14] Berthold HK , Gouni‐Berthold I , Bohm M , Krone W , Bestehorn KP . Patterns and predictors of statin prescription in patients with type 2 diabetes. Cardiovasc Diabetol. 2009;8:25. 19439071 10.1186/1475-2840-8-25 PMC2689197

[15] Jia X , Al Rifai M , Ramsey DJ , et al. Association between lipid testing and statin adherence in the veterans affairs health system. Am J Med. 2019;132(9):e693‐e700. 31103643 10.1016/j.amjmed.2019.04.002

[16] Bhavaraju NN , J. Nanni ; Carlson, C. ; Sholk, J. ; Peterson, K. ; Smith, L. Breaking the Barriers to Specialty Care: Practical Ideas to Improve Health Equity and Reduce Cost. San Francisco, CA: FSG: Reimagining Social Change; 2016.

[17] Hughes‐Cromwick PW , R. Wallace ; Mull, H. ; Bologna, J. Cost Benefit Analysis of Providing Non‐Emergency Medical Transportation. Washington, D.C.: Transportation Research Record; 2005.

[18] Ahmed ST , Mahtta D , Rehman H , et al. Association between frequency of primary care provider visits and evidence‐based statin prescribing and statin adherence: findings from the veterans affairs system. Am Heart J. 2020;221:9‐18. 31896038 10.1016/j.ahj.2019.11.019

[19] Mach F , Baigent C , Catapano AL , et al. 2019 ESC/EAS guidelines for the management of dyslipidaemias: lipid modification to reduce cardiovascular risk. Eur Heart J. 2020;41(1):111‐188. 31504418 10.1093/eurheartj/ehz455

[20] Sidebottom AC , Vacquier MC , Jensen JC , et al. Trends in prevalence of guideline‐based use of lipid‐lowering therapy in a large health system. Clin Cardiol. 2020;43(6):560‐567. 32104922 10.1002/clc.23347 PMC7298995

[21] Rodriguez F , Knowles JW , Maron DJ , Virani SS , Heidenreich PA . Frequency of statin use in patients with low‐density lipoprotein cholesterol >/=190 mg/dl from the veterans affairs health system. Am J Cardiol. 2018;122(5):756‐761. 30055758 10.1016/j.amjcard.2018.05.008 PMC6538561

[22] Singh A , Gupta A , Collins BL , et al. Familial hypercholesterolemia among young adults with myocardial infarction. J Am Coll Cardiol. 2019;73(19):2439‐2450. 31097165 10.1016/j.jacc.2019.02.059

[23] Drozda JP Jr , Ferguson TB Jr , Jneid H , et al. 2015 ACC/AHA focused update of secondary prevention lipid performance measures: a report of the American College of Cardiology/American Heart Association task force on performance measures. J Am Coll Cardiol. 2016;67(5):558‐587. 26698405 10.1016/j.jacc.2015.02.003

[24] Karatasakis A , Danek BA , Karacsonyi J , et al. Effect of PCSK9 inhibitors on clinical outcomes in patients with hypercholesterolemia: a meta‐analysis of 35 randomized controlled trials. J Am Heart Assoc. 2017;6(12):1‐13. 10.1161/JAHA.117.006910 PMC5779013 29223954

[25] Grundy SM , Stone NJ , Bailey AL , et al. 2018 AHA/ACC/AACVPR/AAPA/ABC/ACPM/ADA/AGS/APhA/ASPC/NLA/PCNA guideline on the Management of Blood Cholesterol: a report of the American College of Cardiology/American Heart Association task force on clinical practice guidelines. Circulation. 2019;139(25):e1082‐e1143. 30586774 10.1161/CIR.0000000000000625 PMC7403606

[26] Duell PB , Gidding SS , Andersen RL , et al. Longitudinal low density lipoprotein cholesterol goal achievement and cardiovascular outcomes among adult patients with familial hypercholesterolemia: the CASCADE FH registry. Atherosclerosis. 2019;289:85‐93. 31487564 10.1016/j.atherosclerosis.2019.08.007

[27] Cannon CP , Blazing MA , Giugliano RP , et al. Ezetimibe added to statin therapy after acute coronary syndromes. N Engl J Med. 2015;372(25):2387‐2397. 26039521 10.1056/NEJMoa1410489

[28] Schwartz GG , Steg PG , Szarek M , et al. Alirocumab and cardiovascular outcomes after acute coronary syndrome. N Engl J Med. 2018;379(22):2097‐2107. 30403574 10.1056/NEJMoa1801174

